# Real-time and ultrahigh accuracy image synthesis algorithm for full field of view imaging system

**DOI:** 10.1038/s41598-020-69353-9

**Published:** 2020-07-24

**Authors:** Shu-Bin Liu, Jin-Hui Wang, Rong-Ying Yuan, Wu-Xiang Zhao, Lei Li, Qiong-Hua Wang

**Affiliations:** 1 School of Electronics and Information Engineering, Sichuan University, Chengdu, 610065 China; 2 School of Instrumentation and Optoelectronic Engineering, Beihang University, Beijing, 100191 China

**Keywords:** Optics and photonics, Applied optics, Optical techniques, Other photonics

## Abstract

In this paper, we propose a real time, ultrahigh accuracy and full-field-of-view (RUF) algorithm for full field of view (FOV) imaging system. The proposed algorithm combines rough matching and precise matching method to stitch multiple images with the whole FOV in short time and high imaging quality. In order to verify real-time imaging effect of RUF algorithm, we also fabricate a multi-camera imaging system which includes 19 independent cameras. And the experiment result practically illustrates that full-FOV system can achieve good performances under a near-limiting FOV of 360° × 240° with low distortion, meanwhile, optical resolution reaches up to 95 megapixels. 100% registration-accuracy RUF algorithm for imaging in one second can be widely applied to any optical imaging engineering field with large FOV, such as remote sensing imaging, microscopy imaging, monitoring system engineering fields and so on.

## Introduction

Optical imaging systems are now widely used in medical, biological and military fields^1–3^. With the expansion of application scope, higher performance indexes of imaging systems are required, such as larger FOV, higher resolution and lower distortion. In previous work, the conventional single-channel imaging system adopts a complex diffraction/refraction hybrid system^4^. However, the uncorrected second-order spectrum of the optical glass results in a poor image quality. Some scientists make some contributions to the planar imaging system with multiple channels^5–8^. However, FOV is small and imaging quality is at a low level. Besides, multi-camera imaging system without image processing technology requires multi-window scanning imaging, which causes great confusion to observation for real-time and large-FOV performance^9^. To obtain full FOV and high resolution, usually image synthesis algorithms are needed and the system combined with image synthesis algorithms can obtain high resolution^10–17^. For example, AWARE-2 camera is proposed, which achieves high resolution synthesized image by algorithm. However, it only has horizontal 90°–120° FOVs and cylindrical 60°–70° FOVs, which can’t provide a capacious vision^10^. Furthermore, the conventional image processing algorithms, such as rotation-clipping-mapping algorithm, often contain relatively large errors and the imaging precision is at a millimeter level^11^. Besides, conventional large-FOV imaging system with a pixel-level accuracy can’t guarantee a full field-of-view imaging effect^12^. And High-resolution imaging system based on traditional imaging synthesis algorithm is difficult to realize real-time imaging, which is extremely detrimental to engineering requirements^14^. Some image processing techniques matching imaging system can’t deal with the distortion, which causes bad distortion^15^. The imaging system using scanning method is also time-consuming and make a timely judgment on the imaging result^16^. Also, some imaging systems with image processing technique have no stable robustness and accuracy rate facing complex environment^17^. These conventional multi-channel imaging systems can’t meet the functional requirements of large FOV, high resolution and low distortion due to the limitation of optical principle. Therefore, an imaging system with the large FOV, high resolution and low distortion is highly demanded.

In this paper, we propose a real time, ultrahigh accuracy and full-field-of-view (RUF) algorithm for full field of view (FOV) imaging system. The proposed algorithm combines rough matching and precise matching method to stitch multiple images with the whole FOV in short time and high imaging quality. In order to verify real-time imaging effect of RUF algorithm, we also fabricate a multi-camera imaging system which includes 19 independent low-cost cameras. And the experiment result practically illustrates that full-FOV system can achieve good performances under a near-limiting FOV of 360° × 240° with low distortion, meanwhile, optical resolution reaches up to 95 megapixels. 100% registration-accuracy RUF algorithm for imaging in one second can be widely applied to any optical imaging engineering field with large FOV, such as remote sensing imaging, microscopy imaging, monitoring system engineering fields and so on.

## Theory on the proposed algorithm

### System model

**Figure 1:**
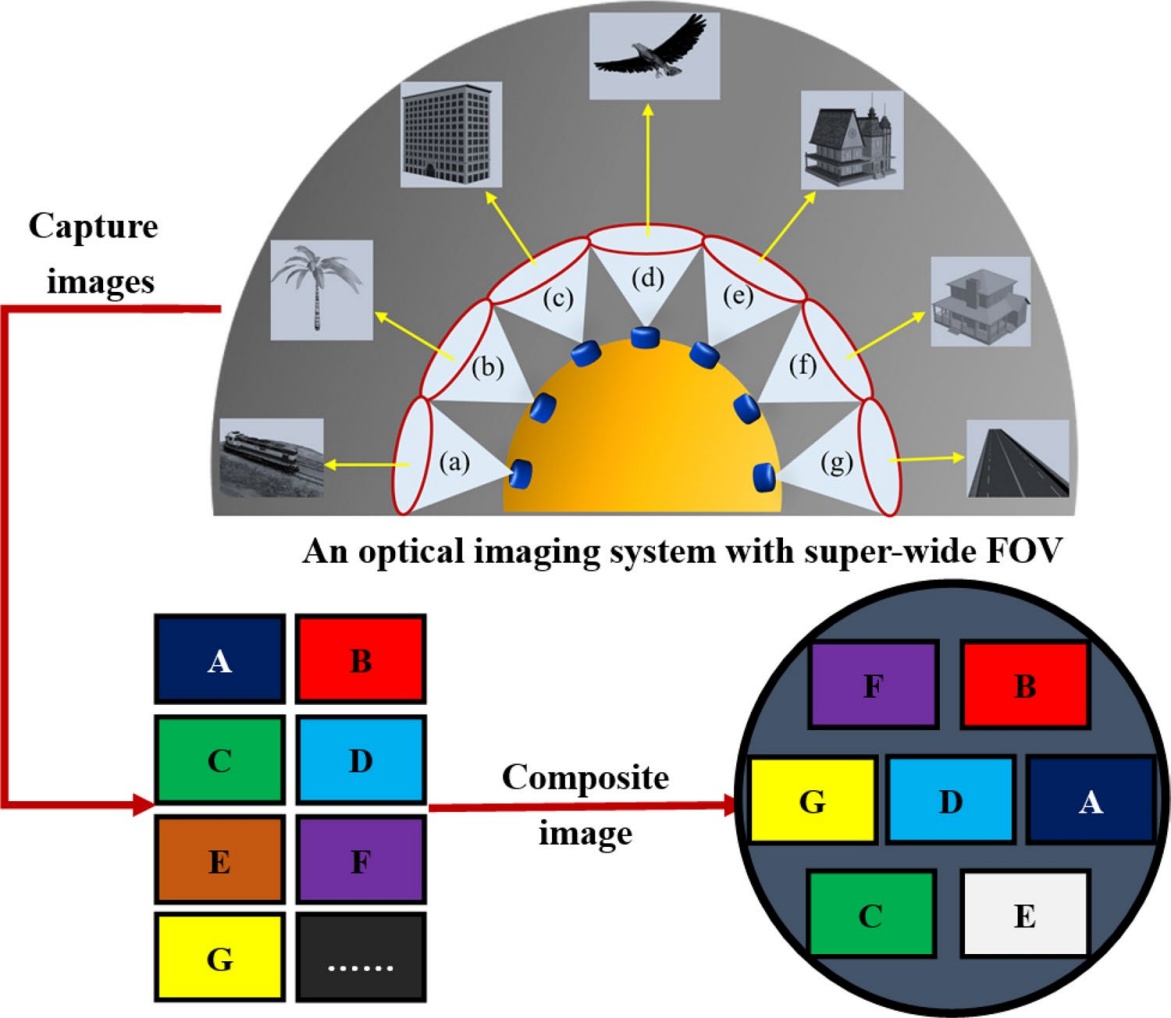
The system model of proposed algorithm.

Figure 1 illustrates the system model of the proposed algorithm, where multi-aperture imaging system divides the whole object space into several small FOVs (for example, a–g), and each imaging channel captures an image (A–G) with a small FOV. And the captured images are mosaicked by our proposed algorithm to get a large-FOV and high-resolution image.

The imaging relationship between the total FOV and a single FOV is obtained via analyzing the imaging principle of large FOV, the total FOV of the imaging system can be expressed as:

1 $$ TFOV = nFOV - F_{C} , $$

where *TFOV* is the total FOV of the imaging system and *FOV* is a single FOV. And n is the number of system apertures, evidently Fc is the FOV of the overlapping portion. Furthermore, there is a theoretical possibility of infinitely scalable field angles and pixel values, but still maintains subject to low-cost camera facilities and wide-FOV principle.

### Real time, ultrahigh accuracy and full-FOV (RUF) algorithm

**Figure 2:**
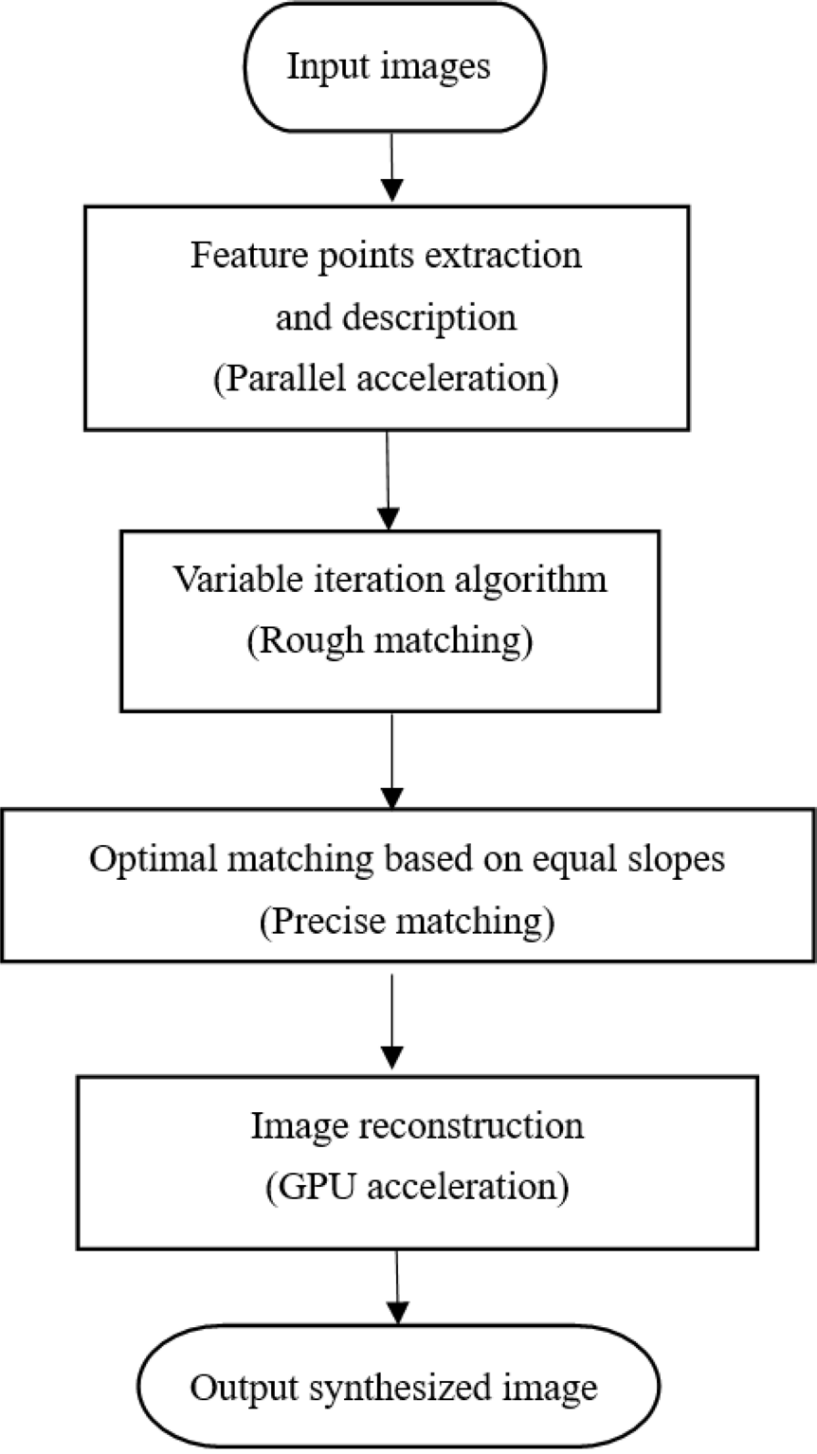
The flowchart for our proposed RUF algorithm.

Based on the above structure, we propose a RUF algorithm for full field of view imaging system. The proposed algorithm combines rough matching and precise matching method. The flowchart for our proposed RUF algorithm is shown in Fig. 2.

In this prototype, multi-camera outputs have the natural advantages of parallel-processing so real-time imaging is realized, which meets practical requirements of real-time and ultrahigh-precision (100%) imaging.

The RUF includes four main steps: parallel acceleration using multithreading and GPU technology, extraction and description of feature points, precision optimization (100% precision) and image reconstruction. Benefiting from independent multi-view snapshot, multi-view real-time imaging is accomplished using multithreading technique. AKAZE^18^ is used to extract feature points, which is implemented using multithreading technology. Feature points in multiple images are extracted independently without affecting each other, so the number of created threads (N) and the number of images (M) satisfy the following relation:

2 $$ Y = \left\{ {\begin{array}{*{20}c} {M - 1,N > M} \\ {N - 1,N < M,} \\ \end{array} } \right. $$

where *Y* is the number of threads that need to be re-created with the exception of the main thread, and a mutex is added to prevent multiple threads from accessing the same image at the same time. Due to the synchronized efficiency, parallel time is significantly less than serial time. Then previous feature points extracted are matched by FREAK descriptors \* MERGEFORMAT 19, RUF combining AKAZE and FREAK yields a RUF feature description.

**Figure 3:**
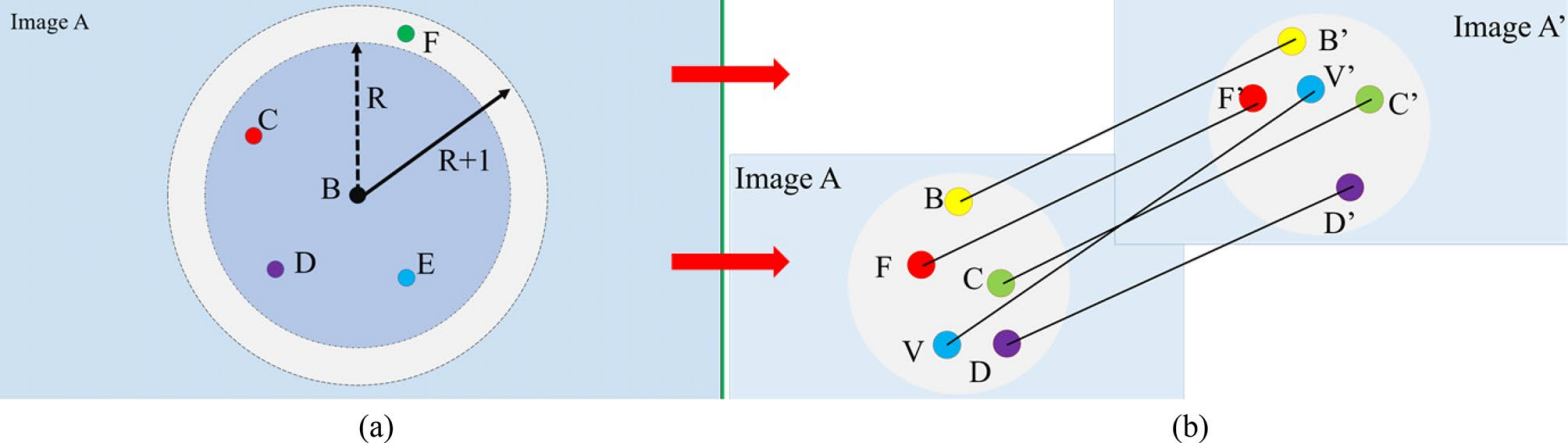
The schematic diagram of accuracy optimization for proposed RUF algorithm. (**a**) The principle of the variable iteration step. (**b**) Optimal matching based on equal slopes.

Precision optimization utilizes two-step method: (1) the proposed variable iteration method for the rough matching. (2) Optimal matching based on equal slopes for the precise matching. We demonstrate it as shown in Fig. 3. In this process, points of interest are matched and the rest of points are discarded, where the best registration result (100% accuracy) is used for following image reconstruction. It is noted that the above two steps all satisfy the symmetry principle. That is, each image and reference image must meet the same condition of feature matching before they will be regarded as good point pairs by default. Conventional matching filtration method usually utilizes the method of constant threshold to judge whether it belongs to a pair of matching points, and only satisfies the requirement of unilateral matching. By constantly changing new fixed threshold, however, some feature matching pairs can be obtained but some error matches still exist (due to the constant threshold). In this paper, we propose a variable iteration algorithm, which aims to solve the problem of mismatches. The theory with our proposed method for the rough filtration is illustrated in Fig. 3a, where the blue rectangle represents the image A, points (B–F) are the points of interest reserved for the primary filtration, blue and white circles are search areas with different radii. In this step, we select a point of interest matched (B) as a center point, which is regarded as a good point of interest with four points (n = 5) around it in a circle of radius R. However, if the center point is only with three points (n < 5) around it, then we need to add one to the radius (R + 1) until the fifth point of interest is found to jump out of the loops, the algorithm core is described as follows:

3 $$ L = \left\{ {\begin{array}{*{20}l} {R,n \ge 5} \\ {R + 1,n < 5,} \\ \end{array} } \right. $$

where *L* represents the circle search length that is a variate, which has good advantage for accuracy filtration over the comparison method. This idea avoids the disadvantage of circle search using constant search radius, which has important implications for ultrahigh-precision imaging. Unideal matching point pairs are removed. The principle of optimal matching based on equal slopes is illustrated in Fig. 3b, where optimal matching based on equal slope is used to reserve the optimal matched point pairs. The expression for the equal slope is as follows:

4 $$ kn = \frac{yn - yn^{\prime}}{{xn - xn^{\prime}}}, $$

where ${k}_{n}\bullet$*kn* is the nth slope of the line that matches the points, and ($xn$, $yn$) is the coordinate of the nth matched points, and ($xn^{\prime}$, $yn^{\prime}$) is the coordinate of the nth’ matched points in the reference image. As illustrated in Fig. 3b, point pairs matched (for example, B and B′, C and C′, D and D′, F and F′) are right to be reserved and the wrong pairs (V and V′) are discarded. Then the mapping between each image and reference image is estimated by perspective transformation. The mapping images are defined in a uniform coordinate system, the relationship is given by –the following relational expression:

5 $$ \left[ {X,Y,Z} \right] = M\left[ {x,y,z} \right], $$

where [*x*, *y*, *z*] is the coordinate of original image and [*X*,* Y*,* Z*] is the coordinate of new image with transformation, *M* is the transformation matrix which helps us to obtain the perspective transformation model preparing for the following image reconstruction.

The obtained registration information is used for the image reconstruction. In the process of reconstruction, we utilize a multi-band fusion and exposure compensation method to output large-FOV and high-resolution image. GPU computing makes this process in parallel processing architecture, which allows research to be conducted not only in technical areas but also to provide assistance for practical engineering needs. In RUF algorithm, GPU parallel processing is already realized, so our proposed RUF algorithm enables to output real-time, ultrahigh-precision image with full-FOV.

## Experiment

### System design and simulation

**Figure 4:**
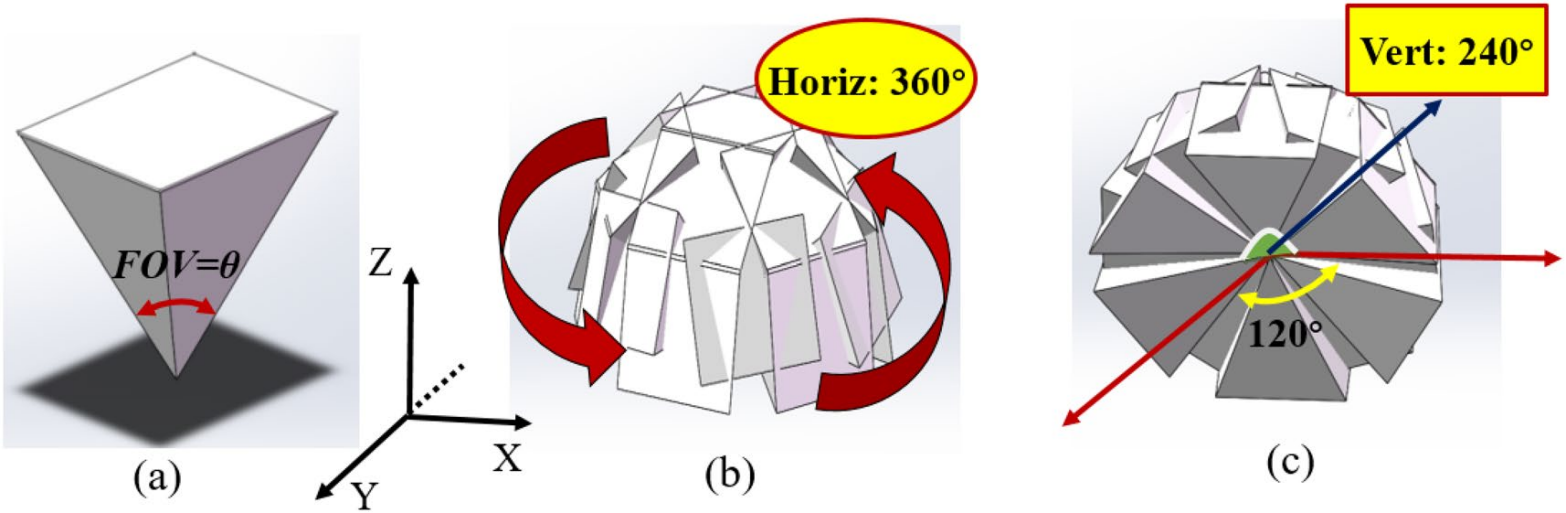
FOV of 19 cameras. (**a**) FOV of a single camera. (**b**)Overlap model of the view 1. (**c**) Overlap model of the view 2.

To get the whole FOV, we first designed and modeled a 19 cameras imaging system are placed in a given position in and simulated the FOVs of the 19 cameras in SolidWorks software platform. Figure 4 shows the FOV model of 19 cameras. Figure 4a shows FOV of a single camera, and 19 small FOVs realize the nondestructive detection without dead-zone, the effective FOV of the camera is 100°(*θ* = 100°). Figure 4b, c are the overlap models of 19 FOVs from the different viewing angle. In the simulation, the fabricated system reaches 360° and the vertical angle reaches more than 240°.

For the global camera layout, we establish a three-dimensional coordinate system to describe the distribution characteristics of 19 imaging channels. Based on SolidWorks software platform, the rotation angles of 19 imaging channels are shown in Table 1. Here *i* represents the number of channels, *θ* is the angle of the imaging channel to the X-axis and *β* is that of the imaging channel to the Z-axis. The imaging channel *A* is the center of 19 channels, which is regarded as a reference. Then we define the rotation angle around it as counterclockwise (clockwise) to negative (positive).

**Table 1:** The angle of 19 imaging channels in space (°).

*i*	*A*	*B*	*C*	*D*	*E*	*F*	*G*	*H*	*I*	*J*
*θ*	0	0	− 60	− 120	− 180	120	60	60	30	0
*β*	0	39.8	39.8	39.8	39.8	39.8	39.8	79.6	69.0	79.6

*i*	*K*	*L*	*M*	*N*	*O*	*P*	*Q*	*R*	*S*	
*θ*	− 30	− 60	− 90	− 120	− 150	− 180	150	120	90	
*β*	69	79.6	69	79.6	69	79.6	69	79.6	69	

### RUF algorithm for full field of view imaging system

**Figure 5:**
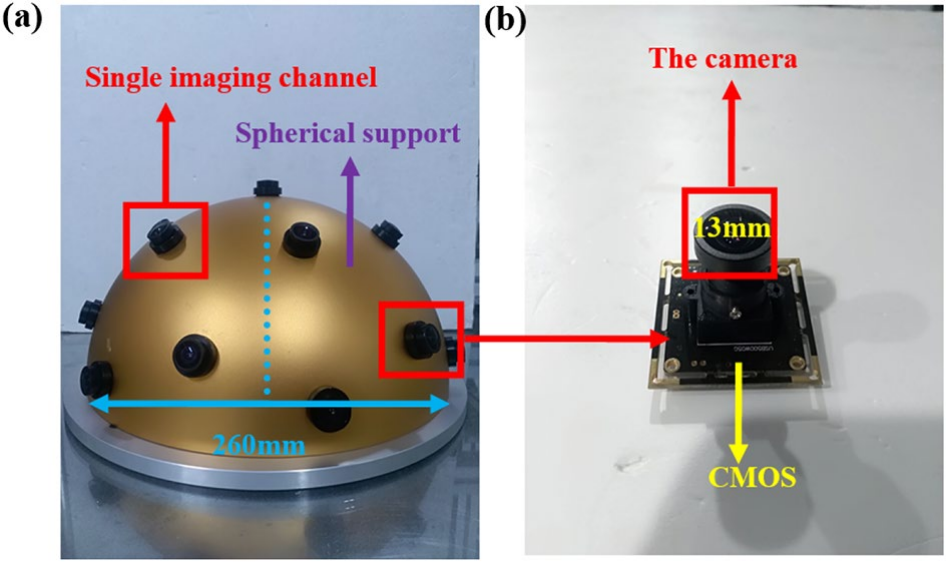
The preparation result of the fabricated system (130 mm × 130 mm × 6 mm). (**a**) The structure of the fabricated system. (**b**) The composition of single imaging channel.

To prove the efficiency of the proposed algorithm, we fabricate a multi-camera imaging system consisting of 19 cameras illustrated in Fig. 5a. The spherical frame is made of aluminum. 19 holes was drilled on it to install 19 cameras. The radius of the frame is ~ 130 mm and the thickness is ~ 6 mm. The camera is shown in Fig. 5b, which consists of a lens and an image sensor. And 13-mm-diameter camera has a FOV of 100° and its focal length is ~ 3.5 mm. Specifically, 19 cameras are used for information capture and the 19 images are captured at 30 frames per second. One computer is used to connect the cameras and storage the data, which is really the hardware carrier used to realize real-time imaging.

**Figure 6:**
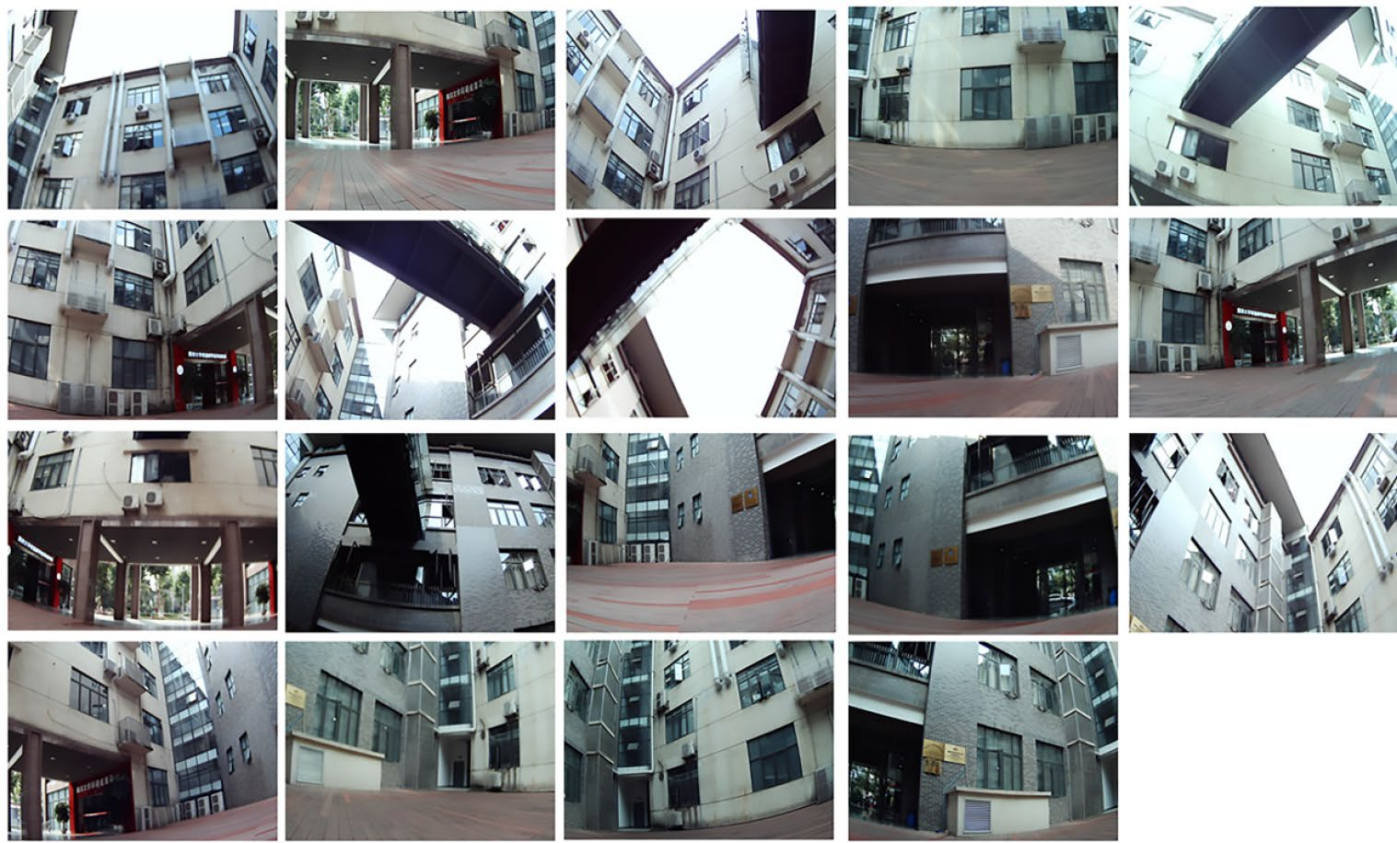
The 19 images (captured on 13 June 2019 at 15:26).

**Figure 7:**
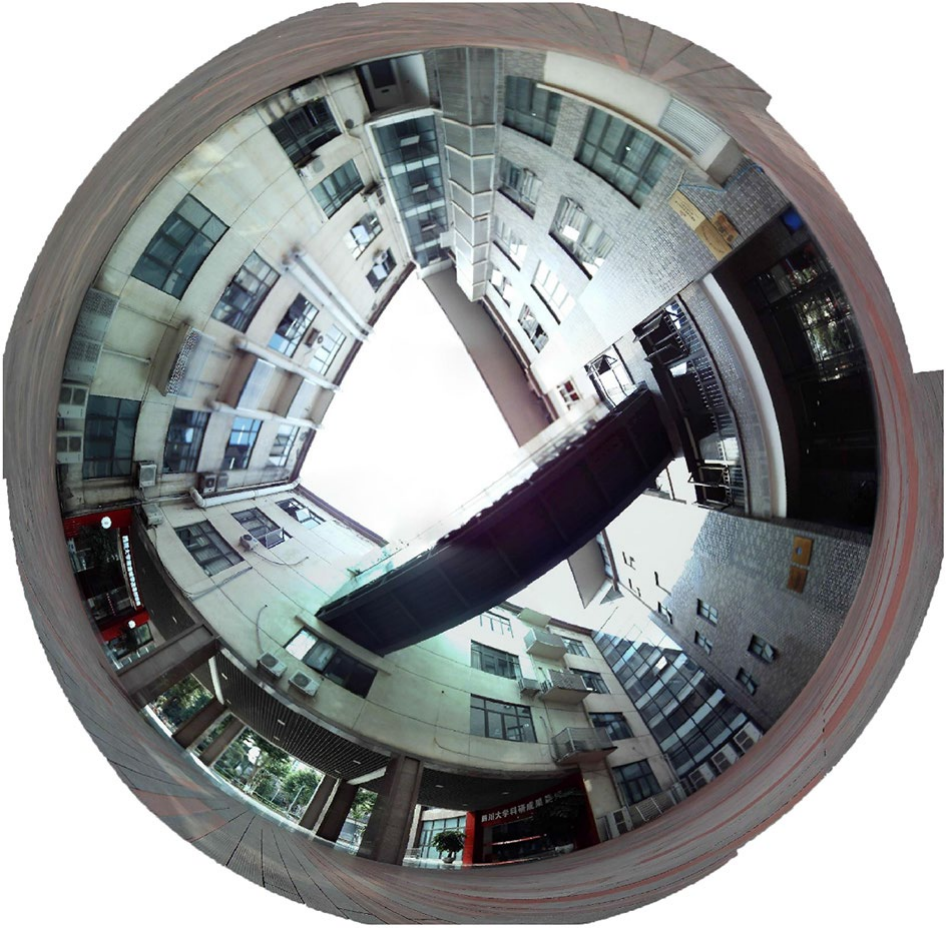
The whole-FOV image captured by fabricated system.

In the experiment, we use the fabricated system to take the image of a building. Firstly, the fabricated system captured the whole building. Then, the 19 images are synthesized using the proposed algorithm. We note that the algorithm is working on a PC (Intel Core i9-9880H CPU @2.3 GHz/4.8 GHz + RTX2080) equipped with Windows10 operating system and is based on the platform of vs2019 + opencv4.2. Each image has a resolution of 2,592 × 1,944. The 19 images are shown in Fig. 6. The whole-FOV image is shown in Fig. 7. From Fig. 7, we see that the synthesized image has large FOV, high resolution and low distortion. In the experiment, the whole FOV of the image is 360° × 240°. We can see each part of the image is very clear, here optical resolution reaches up to 95 megapixels. As an engineering requirement, real-time imaging is yet realized.

**Figure 8:**
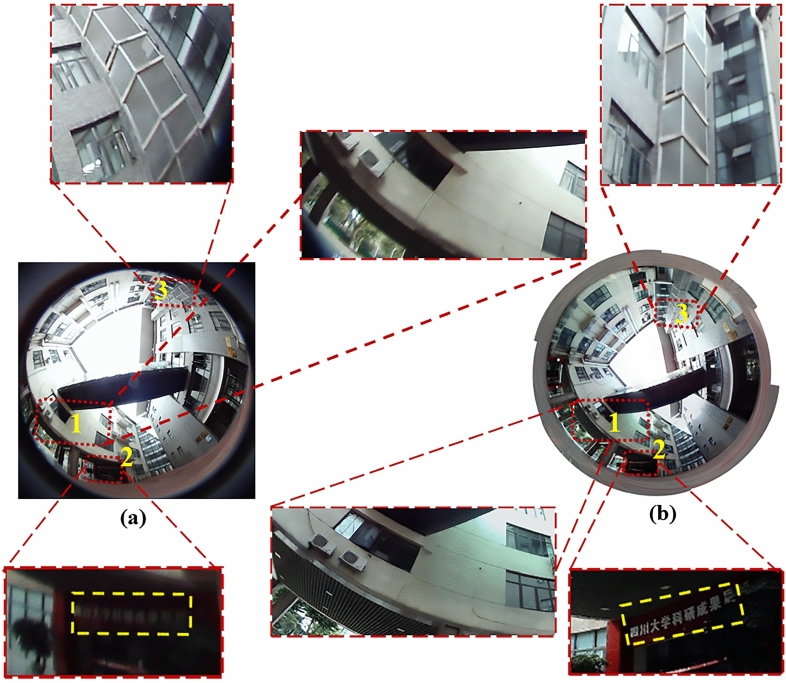
The comparison of the fisheye camera and our fabricated imaging system. (**a**) 360° fisheye image captured by fisheye camera:the regions 1–3 are the enlarged details of the labelled regions in (**a**). (**b**) The synthesized image with large FOV, high resolution and low distortion: images 1–3 are the enlarged details of the labelled regions in (**b**).

We also compare the fabricated system with a conventional fisheye camera with 5-megapixel as shown in Fig. 8. Both systems can obtain a large-FOV image. However, if we compare the details, the performances of the two systems are different.

Comparing the details of the two images in region 1, the fabricated system demonstrates that part of scene from the bridge bottom to the grille ceiling. It provides accurate information, for example, exactly how many linear grilles (50) are between the 2 elongated white boards on the building ceiling. Also, exactly how many grilles (3) are occupied by one white board, which is also clear to see the details of window on the wall. However, it’s unable to see clearly using the fisheye camera.

Comparing the details of the two images in region 2, the conventional fisheye camera is unable to take the image of the whole scene with uniform exposure. For example, some parts of the scene may be overexposed, and the other parts of the scene may be underexposed due to complex lighting environment in real world. However, the fabricated system avoids this problem. The fabricated system shows an example high-dynamic-range (HDR) image, the sunlight of that varies from bright wall to the dark ceiling and returns to the colorful scene, which is difficult to get uniform exposure using the fisheye camera (see region 2 in fisheye image). From the labeled-yellow region in the fabricated system, we can see characters in the exhibition hall clearly. The comparison illustrates an HDR synthesized image without the partial darkness covers the color information of the object space. This difference mainly benefits from independent exposure of each camera, which more accurately matches human vision.

Comparing the details of the two images in region 3, the conventional imaging system has serious distortion due to large FOV. However, the proposed algorithm synthesizes multiple low-dynamic-range (LDR) images into an HDR image. Each camera has relatively small FOV, which largely reduces the distortion. For instance, the white border of cuboid building in the synthesized image is clearly straight while that in the fisheye image is curved. A combination of system and algorithm provides a competitive advantage over the comparison system. Therefore, our fabricated system can get large-FOV image with low-distortion and high-resolution.

### Accuracy of RUF

To prove the accuracy of the proposed algorithm, we did some experiments and illustrated the whole imaging process of RUF image synthesis algorithm.

**Figure 9:**
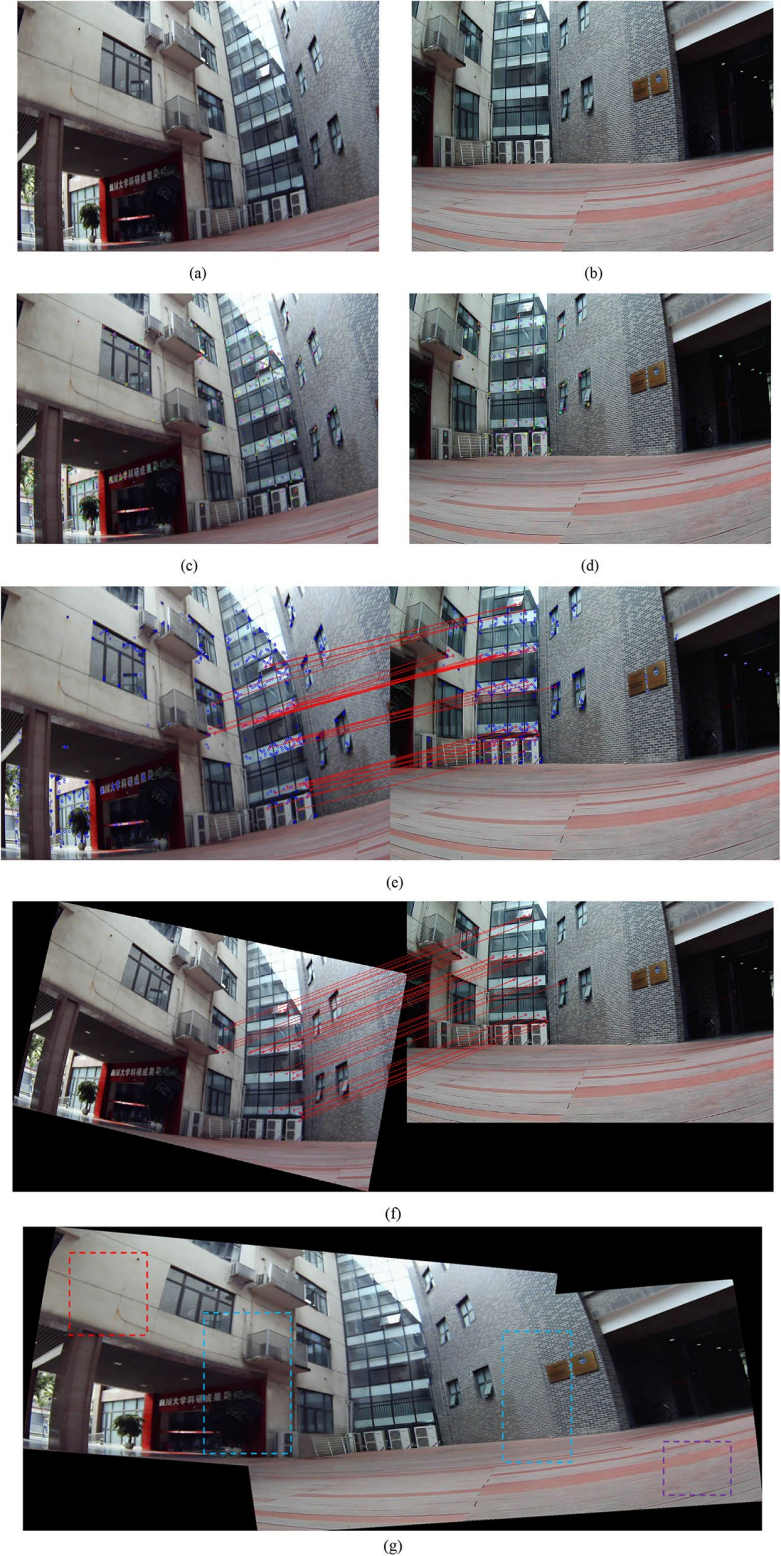
A near-field scene for RUF imaging, (**a**,**b**) original images, (**c**,**d**) points of interest are extracted, (**e**) variable iteration method for the rough matching, (**f**) optimal matching for the precise matching, (**g**) the result for image reconstruction.

An example for points of interest detection-filtration-matching is illustrated in Fig. 9a–g. Figure 9a, b are the input images, Fig. 9c, d show that feature points are marked using circles with various colors. Our proposed variable iteration method is used to remove the bad points of interest, and the matching result is illustrated in Fig. 9e, where the blue points of interest are discarded and the red are matched as good matches.

Due to the introduction of variable thresholds, the matching precision is improved a lot until the good matches are retained (see previous “RUF algorithm for full field of view imaging system” section for details). Considering the computation, the optimal matches based on equal slopes is expected (see previous “RUF algorithm for full field of view imaging system” section for details). The matched result is illustrated in Fig. 9f, the red matched pairs with equal slopes are reserved and used for finding the optimal solution. The result for image reproduction is illustrated in Fig. 9g, which proves that the RUF method has the ability to adapt to complex environment including scale scaling, uneven illumination and rotation. RUF enables real-time, 100% accuracy and high-resolution imaging to be relized.

**Figure 10:**
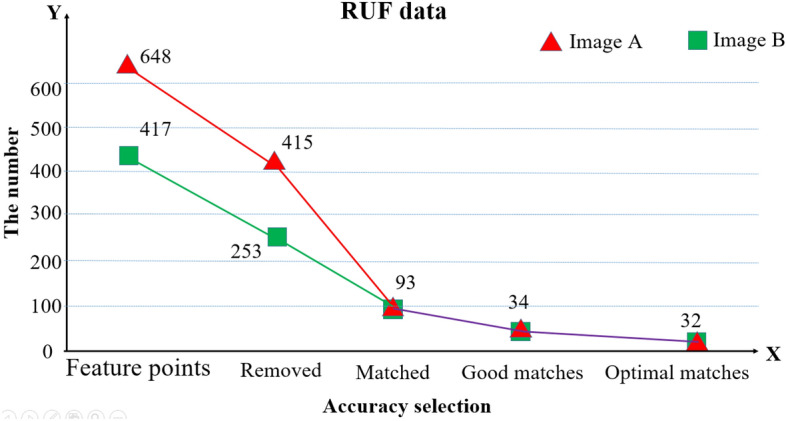
The line chart of RUF data.

As illustrated in Fig. 10, the RUF example data is given. In this step, GPU acceleration and multithread technology enables real-time imaging to be possiable, and the matched pairs based on equal slopes can always be found to be the best solution, Fig. 10 illustrates RUF registration information achieves 100% accuracy in a near-field scene. We introduce SSIM^20^ to quantitatively evaluate image quality. We obtain 2 blue regions of the patchwork areas in the composite picture (see Fig. 9g for blue regions). As illustrated in Table 2, the values of SSIM1 (comparing the left-blue region with identical region in original image a) and SSIM2 (comparing the right-blue region with identical region in original image b) are computed. Image synthesis algorithm is able to adapt to complex environments. Previous sections have proved the adaptability of RUF algorithm to angle, light and scale, different rotation angles, and light conditions also cause SSIM to drop. This decline is predictable and acceptable. The red and purple regions are selected to operate the further verification, Table 2 shows the SSIM value close to split seam can be stable above 0.95 and non-stitched region can be stable above 0.98. The results demonstrate that image quality is clearly improved (SSIM_max_ = 1). This is because that both images are from the original image (theoretically, SSIM = 1). Due to the distortion, rotation, scaling and other performance effects of the algorithm for the synthesized image, the measured SSIM value must be less than 1, but the stitching accuracy is infinitely close to 100%.

**Table 2:** The image quality evaluation for near-field scene.

	Registration (%)	Reconstruction (SSIM_1,2,3,4_)	Average (SSIM_1,2,3,4_)
Left-blue image	100	0.972	0.962
Right-blue image	100	0.953	0.962
Left-red image	100	0.974	0.984
Right-purple image	100	0.995	0.984

**Figure 11:**
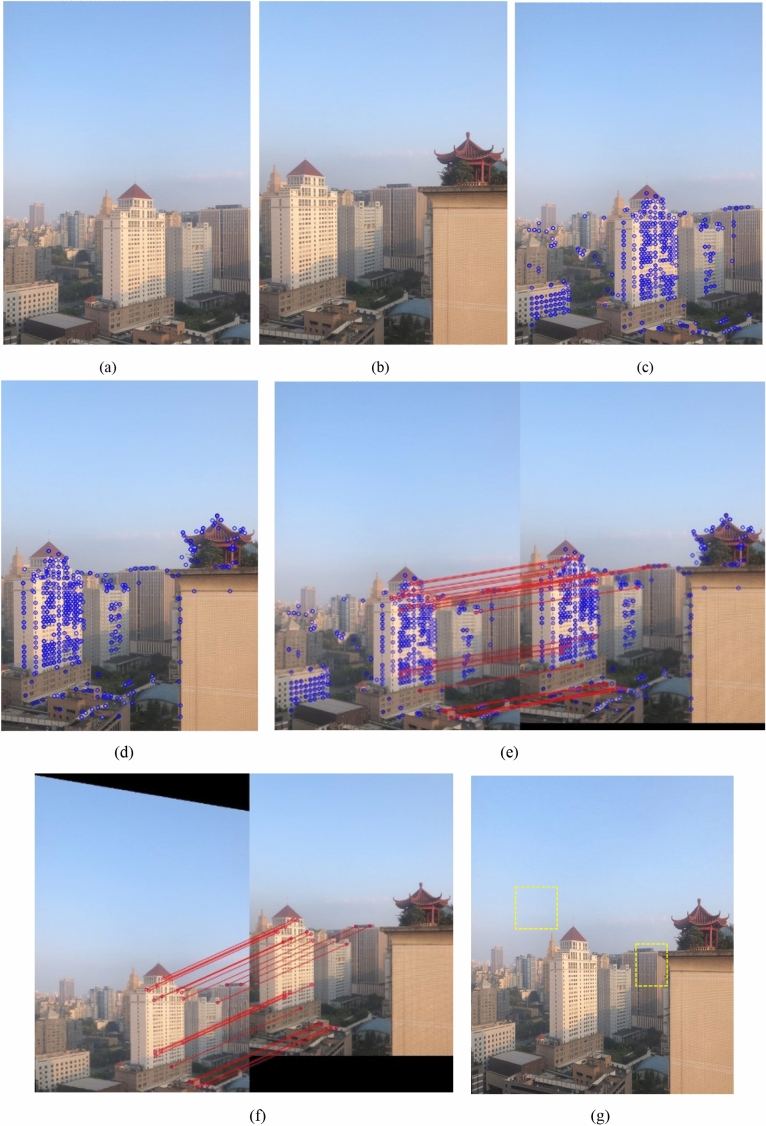
A far-field scene for RUF imaging, (**a**,**b**) original images, (**c**,**d**) points of interest are extracted, (**e**) variable iteration method for the rough matching, (**f**) optimal matching for the precise matching, (**g**) the result for image reconstruction.

In order to further prove the ultrahigh accuracy of the algorithm, a far-field scene is illustrated in Fig. 11. Figure 11 shows all points of interest are right (100% registration rate). SSIM is introduced to evaluate the image quality, and Fig. 11g demonstrates SSIM indexes of 2 yellow-regions near the seam all are 1.0. Therefore, the proposed algorithm with real-time imaging can achieve ultrahigh accuracy (100%), which is of great significance for the application of algorithms in engineering.

The processing time of RUF for full-FOV system is described in Table 3. Table 3 shows the required time for each step of full-FOV algorithm (for full-FOV system: 19 images). Table 4 demonstrates the detailed processing time for various number of images (2, 7, and 19), where near-field and far-field scenes correspond to 227 ms and 158 ms respectively. Comparing with serial time, parallel processing improves by an order of magnitude.

**Table 3:** The time of algorithm step for full-field-of-view imaging system.

Step	Feature extraction	Accuracy optimization	Image construction
Time	218 ms	230 ms	534 ms

**Table 4:** The detailed processing time for various number of images.

Numbers	2 images	7 images	19 images	Without parallel
Time	227 ms/158 ms	478 ms	1002 ms	14034 ms

## Conclusion

In this paper, we propose a real time, ultrahigh accuracy and full-field-of-view (RUF) algorithm for full field of view (FOV) imaging system. The proposed algorithm combines rough matching and precise matching method to stitch multiple images with the whole FOV in short time and high imaging quality. In order to verify real-time imaging effect of RUF algorithm, we also fabricate a multi-camera imaging system which includes 19 independent cameras. And the experiment result practically illustrates that full-FOV system can achieve good performances under a near-limiting FOV of 360° × 240° with low distortion, meanwhile, optical resolution reaches up to 95 megapixels. 100% registration-accuracy RUF algorithm for imaging in one second can be widely applied to any optical imaging engineering field with large FOV, such as remote sensing imaging, microscopy imaging, monitoring system engineering fields and so on.

## Appendix

We utilize *SSIM* as quantitative evaluators in this paper. The *SSIM* is defined as:

6 $$ SSIM(x,y) = \frac{{\left( {2u_{x} u_{y} + c_{1} } \right)\left( {2\sigma_{xy} + c_{2} } \right)}}{{\left( {u_{x}^{2} + u_{y}^{2} + c_{1} } \right)\left( {\sigma_{x}^{2} + \sigma_{y}^{2} + c_{2} } \right)}}, $$

7 $$ c_{1} = \left( {k_{1} L} \right)^{2} ,c_{2} = \left( {k_{2} L} \right)^{2} , $$

where $u_{x}$ and $u_{{\text{y}}}$ is the average of *x* and *y*, $\sigma_{{}}$is variance and $\sigma_{{{\text{xy}}}}$ is covariance of *x* and *y*, *L* is the dynamic range of the pixel values, and $k_{1} = 0.01$, $k_{2} = 0.03$ by default. The ideal value of *SSIM* is in the range between ‘0’ to ‘1’, where *SSIM *= 1 means two identical images completely.
